# Effects of electrostatic environment on the electrically triggered production of entangled photon pairs from droplet epitaxial quantum dots

**DOI:** 10.1038/s41598-018-38044-x

**Published:** 2019-02-07

**Authors:** Hanz Y. Ramírez, Ying-Lin Chou, Shun-Jen Cheng

**Affiliations:** 1 Escuela de Física, Universidad Pedagógica y Tecnológica de Colombia (UPTC), Tunja, 150003 Boyacá Colombia; 2 Department of Electrophysics, National Chiao Tung University (NCTU), Hsinchu, 30050 Taiwan Republic of China

## Abstract

Entangled photon pair generation is a crucial task for development of quantum information based technologies, and production of entangled pairs by biexciton cascade decays in semiconductor quantum dots is so far one of the most advanced techniques to achieve it. However, its scalability toward massive implementation requires further understanding and better tuning mechanisms to suppress the fine structure splitting between polarized exciton states, which persists as a major obstacle for entanglement generation from most quantum dot samples. In this work, the influence of electrostatic environment arising from electrically biased electrodes and/or charged impurities on the fine structure splitting of GaAs/AlGaAs droplet epitaxial quantum dots is studied, by means of numerical simulations considering a realistic quantum dot confining potential and electron-hole exchange interaction within a multiband *k* · *p* framework. We find that reduction of the fine structure splitting can be substantially optimized by tilting the field and seeding impurities along the droplet elongation axis. Furthermore, our results provide evidence of how the presence of charged impurities and in-plane bias components, may account for different degrees of splitting manipulation in dots with similar shape, size and growth conditions.

## Introduction

**Figure 1:**
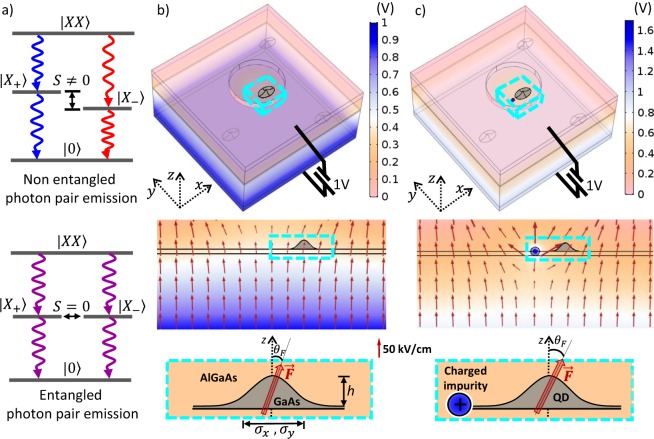
(**a**) Schematics of the biexciton-exciton-vacuum (*XX* − *X* − 0) cascade decay processes emitting polarized photon pairs which are non-entangled with *S* ≠ 0 (top panel) and quantum-entangled with *S* = 0 (bottom panel). (**b**) Top panel: Simulated electrostatic potential distribution in an electrically gated semiconductor where QDs are embedded. Middle panel: The electric field distribution on the cross section of the plane *y* = 0 between a top gating electrode and a grounded bottom one which are separated by 200 nm and biased by a voltage *V* = 1 V. For collecting the vertically emitted photons from the QDs underneath, a circular aperture is created on the top electrode and leads to the inhomogeneity of electric field distribution. Bottom panel: Zoom of the non-central region in the aperture where a QD is subjected to a tilted electrical field. (**c**) same as in (**b**) but in addition of a charged impurity near to the QD.

Quantum dots (QDs) have been demonstrated as promising high quality photon sources, vital for optoelectronic applications in optical quantum technologies, such as quantum teleportation and quantum cryptography^1–4^. In particular, control of the exciton fine structure splitting (FSS) has become a widely studied problem in solid state physics of zero dimensional systems, owning to its crucial role in production of polarization -entangled photon pairs^5–9^. Nevertheless, in spite of significant developments, choosing suitable QDs for optimal restoration of the polarized exciton degeneracy is yet a “cherry picking” process, and some important issues remain to be addressed considering that cutting edge utilizations require operatively efficient and scalable FSS tuning. Figure 1(a) presents a schematics of the biexciton-exciton-vacuum cascade process through which an entangled photon pair could be generated, without the need of post-selection processes^10^, as long as the FSS can be tuned to be sufficiently small, comparable with or even smaller than the intrinsic line width of emitted photons that is typically ~1 *μ*eV.

Electrical control and suppression of the FSS of QDs are two essential issues, besides coherence and optical brightness^11,12^, in the development of QD-based entangled photon pair emitters that can be integrated in semiconductor microelectronic systems and scalable for extending the scope of current photon-based quantum applications^13–15^. Toward realization of such quantum light sources, attempts to tune the FSSs of QDs have been made by fabricating them in semiconductor p-n junctions biased by electrodes in lateral^16–18^, or vertical^19–21^ configurations. For the sake of compatibility with the current epi-layer structure of integrated microelectronic systems, QDs under vertical bias control attracts special attention. However, the use of vertical bias was not demonstrated as a feasible means to control and fully suppress the FSSs of QDs for the entangled photon pair generation until in 2010 Bennett *et al*. fabricated the QD devices in a Schottky architecture that can sustain ultra-high vertical fields and successfully demonstrated the production of entangled photon pairs via vertical bias control^22^. Since then, some improvements and new applications have been achieved for electrically tuned FSS through field applied along the QD growth direction^23,24^. Nonetheless, the inability of effectively reducing the splitting energy in most QDs persists and motivates further research.

Two puzzling questions are addressed in this work: why the level of control on the FSS differs significantly between QDs of similar sizes and compositions? and how vertical field may suppress the splitting in despite of acting perpendicularly to the plane in which the wave function asymmetry crucially underlies it?

We shall show that both issues are closely related to the electrostatic background which impacts differently each randomly positioned QD. The electrostatic effect on a QD is featured by the strength and direction of the electric fields locally penetrating the dot (which is actually slanted in most stacked cases, as will be discussed below), in addition to the modification of the QD confining potential made by surrounding charged defects or impurities, which have been recently identified to a great extent, as the major cause of optical spectral diffusion and dephasing^25–27^.

On the other hand, droplet epitaxy has emerged as one of the most promising techniques to controllably fabricate semiconductor quantum dots (QD)s^7,8^, and increasing publications reporting on their interesting and potentially useful optical properties, make understanding of emission features from single droplets, a relevant problem. In particular, droplet epitaxial (DE) GaAs/AlGaAs QDs serve as an excellent test bed for studying the bias-controlled optical properties of zero dimensional structures, given the convenient removal of strain and technically controllable geometries achieved with this technique^28–31^.

This work presents a theoretical and computational investigation within a multiband framework, on the FSS of single exciton states in droplet epitaxial GaAs/AlGaAs QDs under environmental electrostatic elements such as tilted fields and surrounding impurities. Our studies identify the essential role of horizontal electric-field component from bias and neighboring charges in reshaping the in-plane carrier wave functions, which is, under certain conditions, helpful for the enhanced control of polarized photons from emitting DE-QDs. The paper consists of three main parts. First, we describe the theoretical model in which exciton energies, wave functions and FSSs are computed. Then, the numerical results for different tilting angles and nearby charge positions are shown. Pertinent discussion and conclusions are drawn at the end.

## Theoretical and Computational Methodology

### Single particle spectra of QDs

We begin with calculations for the single particle energy spectra and wave functions of GaAs DE-QDs under external electric fields. The external electric fields imposed onto a QD could arise from the electrostatic environment, including the biased electrodes patterned on the surfaces of QD samples or charged impurities resident around the QDs. Taking advantage of the wide band-gap of GaAs, in this study the single-band effective mass and the Luttinger-Kohn four-band *k* · *p* models are separately used to solve the energy spectra and wave functions of single conduction electrons and single valence holes confined in GaAs DE-QDs^32–35^.

In the single-band context, the wave function of a single conduction electron in a QD is written as ${\psi }_{{i}_{e}}^{e}({\overrightarrow{r}}_{e})={g}_{{i}_{e}}^{e}(\overrightarrow{r}){u}_{{s}_{z}}^{e}({\overrightarrow{r}}_{e})$, a direct product of the smoothly varying envelope function, ${g}_{{i}_{e}}^{e}({\overrightarrow{r}}_{e})$, and the microscopic Bloch function of conduction electron, ${u}_{{s}_{z}}^{e}({\overrightarrow{r}}_{e})$, where ${i}_{e}=({n}_{e},{s}_{e})$ stands for the set of indexes, comprising the orbital index *n*_*e*_ = 1, 2, 3, … and the spin one ${s}_{e}=\uparrow /\downarrow$ that indicates the up/down electron spin with *z*-component, ${s}_{z}=+\,\frac{1}{2}/-\,\frac{1}{2}$. The envelope wave function of electron is governed by the single-band Schrödinger equation, ${H}_{e}|{g}_{{i}_{e}}^{e}\rangle ={E}_{{i}_{e}}^{e}|{g}_{{i}_{e}}^{e}\rangle$, with the single-electron Hamiltonian of an electrically biased QD in the absence of charged defects or impurities given by

1 $${H}_{e}=\frac{-\,{\hslash }^{2}\,{\nabla }^{2}}{2{m}_{0}{m}^{\ast }}+{V}_{QD}^{e}({\overrightarrow{r}}_{e})+e\overrightarrow{F}\cdot {\overrightarrow{r}}_{e},$$

where ${\overrightarrow{r}}_{e}=({x}_{e},{y}_{e},{z}_{e})$ is the electron position, ${\nabla }^{2}\equiv \frac{{\partial }^{2}}{\partial {x}^{2}}+\frac{{\partial }^{2}}{\partial {y}^{2}}+\frac{{\partial }^{2}}{\partial {z}^{2}}$, ${V}_{QD}^{e}({\overrightarrow{r}}_{e})$ is the QD confining potential for the electron, ${m}_{e}^{\ast }=0.067$ is the ratio of the GaAs electron effective mass to that of free electron *m*_0_^36^, *e*(>0) is the magnitude of the elementary charge, and $\overrightarrow{F}=({F}_{x},{F}_{y},{F}_{z})$ is the external electric field.

The confining potential of a GaAs/AlGaAs QD for a carrier (a conduction electron or a valence hole) can be expressed, in terms of the band edge difference between GaAs and AlGaAs and the characteristic function of the QD, as ${V}_{QD}^{\nu }({\overrightarrow{r}}_{\nu })={V}_{b}^{\nu }\cdot (1-{X}_{QD}({\overrightarrow{r}}_{\nu }))$, where the superscript *ν* = *e*/*h* denotes electron/hole. A conduction-band (valence-band) offset ${V}_{b}^{e}=300$ meV (${V}_{b}^{h}=200$ meV) is taken for GaAs/AlGaAs heterostructure. According to the observations of atomic force microscope for DE-QDs^27,28,37^, the characteristic function of GaAs DE-QD is modeled (as depicted by Fig. 1(b,c)) by

2 $$X(\overrightarrow{r})=\{\begin{array}{cc}1, & 0\le z\le h\times \exp \,(\,-\,{\textstyle \tfrac{{x}^{2}}{{{\rm{\Lambda }}}_{x}^{2}}}-{\textstyle \tfrac{{y}^{2}}{{{\rm{\Lambda }}}_{y}^{2}}})\\ 0, & \,\,\,{\rm{e}}{\rm{l}}{\rm{s}}{\rm{e}}{\rm{w}}{\rm{h}}{\rm{e}}{\rm{r}}{\rm{e}}\end{array},$$

where *h* is the QD height and Λ_*x*_ (Λ_*y*_) is defined as the characteristic side length of QD in the *x*-direction (*y*-direction). Throughout this work, without loss of generality, we consider a *x*-elongated GaAs/AlGaAs QD with Λ_*x*_ = 12 nm, Λ_*y*_ = 10 nm, and *h* = 12 nm in the simulations, according to the atomic-force-microscope (AFM) images of GaAs DE-QDs on (001) substrates^28^.

To verify the electric field experienced by a QD in a Schottky architecture of photon source device, we carry out numerical finite-element simulations by using the Comsol Multiphysics package^34,38^ to solve the Laplace’s equation (the Poisson equation, for the case in which a charged impurity is also considered) for a QD film sandwiched by top and bottom metallic electrodes separated by 200 nm and subject to electrical bias *V* = 1 V, and a circular aperture of diameter 150 nm is created on the top electrode layer for light emission and collection, as depicted in Fig. 1(b,c). For a QD located in the area of aperture with the applied bias *V* = 1 V, the magnitude and tilting angle of the experienced electric field by the QD is shown to be in the range of *F* = 35–65 kV/cm and *θ* = 0° − 20°, depending on the precise location of QD. Though the electric field induced by the biased electrode is spatially varying, it is verified from the simulation that the spatial variation of electric field through a QD can be neglected because of its small size.

It is worth noting that the structures and sizes of the devices in Fig. 1(b,c) are so chosen for the simplicity of simulation and easy visualization, and might be oversimplified as compared with the practically working devices in experiments^22,23^. Nevertheless, any experimental setup that produces electric fields in the scale of strength as simulated in Fig. 1(b,c) would exhibit analogous physical features. The simulated fields, about tens of kV/cm nearby the QDs, are indeed in the range of the electric fields producible in the existing experiments^22,23,39,40^.

The wave function of a valence hole in a QD described in the four-band model is composed of two heavy-hole components and two light-hole ones^41–44^, as written by ${\psi }_{{i}_{h}}^{h}(\overrightarrow{r})={\sum }_{{j}_{z}=\pm \frac{1}{2},\pm \frac{3}{2}}\,{g}_{{i}_{h},{j}_{z}}^{h}(\overrightarrow{r}){u}_{{j}_{z}}^{h}(\overrightarrow{r})$, where ${j}_{z}=\pm \,\frac{1}{2},\pm \,\frac{3}{2}$ denotes the four possible *z*-components of the pseudo-spin (*J* = 3/2) of hole, ${g}_{{i}_{h},{j}_{z}}^{h}(\overrightarrow{r})$ is the envelope wave function of the *j*_*z*_ component in the *i*_*h*_-th hole state, the subscript *i*_*h*_ = (*n*_*h*_, *χ*_*h*_) is the composite index that comprises the label of hole orbital state, *n*_*h*_ = 1, 2, 3, …, and the symbol $\chi =\Uparrow /\Downarrow$ that indicates the leading heavy-hole component of ${j}_{z}=+\,\frac{3}{2}/-\,\frac{3}{2}$ in the hole state. In the basis ordered by $\{|{u}_{\tfrac{3}{2}}\rangle ,|{u}_{\tfrac{1}{2}}\rangle ,|{u}_{-\tfrac{1}{2}}\rangle ,|{u}_{-\tfrac{3}{2}}\rangle \}$, the Hamiltonian for a single hole in a QD is formulated as a 4 × 4 matrix that can be divided into the kinetic energy-, potential, and external-field parts, ${H}_{h}={H}_{k}^{h}+{H}_{QD}^{h}+{H}_{F}^{h}$. The kinetic part of the hole Hamiltonian is given by

3 $${H}_{k}^{h}=(\begin{array}{cccc}{\mathbb{P}}+{\mathbb{Q}} & -\,{\mathbb{S}} & {\mathbb{R}} & 0\\ -\,{{\mathbb{S}}}^{\ast } & {\mathbb{P}}-{\mathbb{Q}} & 0 & {\mathbb{R}}\\ {{\mathbb{R}}}^{\ast } & 0 & {\mathbb{P}}-{\mathbb{Q}} & {\mathbb{S}}\\ 0 & {{\mathbb{R}}}^{\ast } & {{\mathbb{S}}}^{\ast } & {\mathbb{P}}+{\mathbb{Q}}\end{array}),$$

where ${\mathbb{P}}=-\,\frac{{\hslash }^{2}{\gamma }_{1}}{2{m}_{0}}{\nabla }^{2}$, ${\mathbb{Q}}=\frac{{\hslash }^{2}{\gamma }_{2}}{2{m}_{0}}(\,-\,{\nabla }^{2}+3\frac{{\partial }^{2}}{\partial {z}^{2}})$, ${\mathbb{R}}=\frac{{\hslash }^{2}}{2{m}_{0}}[\,-\,\sqrt{3}{\gamma }_{3}(\frac{{\partial }^{2}}{\partial {y}^{2}}-\frac{{\partial }^{2}}{\partial {x}^{2}})-i2\sqrt{3}{\gamma }_{2}\frac{\partial }{\partial x}\frac{\partial }{\partial y}]$, and ${\mathbb{S}}=\frac{{\hslash }^{2}{\gamma }_{3}}{2{m}_{0}}$ $[i\sqrt{3}(i\frac{\partial }{\partial x}+\frac{\partial }{\partial y})\frac{\partial }{\partial z}]$, and the Luttinger parameters *γ*_1_ = 6.85, *γ*_2_ = 2.1, and *γ*_3_ = 2.9 are taken for GaAs^28,32,36,45^.

Taking the fact that the QD confining potential is slowly varied in space with respect to the microscopic Bloch functions, the Hamiltonian of confining potential for the hole is written in the form of a diagonalized matrix as given by ${H}_{QD}^{h}={V}_{QD}^{h}({\overrightarrow{r}}_{h}){I}_{4\times 4}$, where *I*_4×4_ is the 4 × 4 identity matrix. Likewise, for a slowly varying electric field, the yielded Hamiltonian to a positively-charged hole in a QD reads ${H}_{F}^{h}=-\,e\overrightarrow{F}\cdot {\overrightarrow{r}}_{h}{I}_{4\times 4}$. The energies and wave functions of a single hole in the QD are obtained by numerically solving, ${H}_{h}|{\psi }_{{i}_{h}}^{h}\rangle ={E}_{{i}_{h}}^{h}|{\psi }_{{i}_{h}}^{h}\rangle$, using the finite-difference method^46,47^.

In presence of a charged defect or impurity, we consider the electrostatic potentials induced by the point charge, ${V}_{{\rm{imp}}}^{\nu }$, on both electron and hole (*ν* = *e* and *h*) in the envelop function approximation, and directly attach their potential Hamiltonians to the single-electron and single-hole ones, Eqs 1 and 3, respectively. Without losing generality, throughout this work we consider a positively charged impurity located in the same plane of the QD, and the resulting potential Hamiltonian for electron and hole are, ${V}_{{\rm{imp}}}^{e}=-\,{e}^{2}/(4\pi {\varepsilon }_{0}{\varepsilon }_{b}|{\overrightarrow{r}}_{e}-{\overrightarrow{r}}_{{\rm{imp}}}|)$ and ${V}_{{\rm{imp}}}^{h}={e}^{2}/(4\pi {\varepsilon }_{0}{\varepsilon }_{b}|{\overrightarrow{r}}_{h}-{\overrightarrow{r}}_{{\rm{imp}}}|)\times {I}_{4\times 4}$, where *ε*_0_ is vacuum permittivity and *ε*_*b*_ = 12.9 is the dielectric constant for GaAs.

### Fine structure splitting of exciton

Next, we solve the energy spectra of single excitons in QDs, which are subject to strong electron-hole (*e*-*h*) direct Coulomb interactions as well as *e*-*h* exchange ones^48–52^. Because of the strong quantum confinement, we assume that the wave functions of the lowest bright-exciton states of a QD subject to the spin-irrelevant direct Coulomb interaction are the product of the lowest electron and hole states^53–55^, denoted for brevity of notation, simply by the spin indexes of the lowest electron and hole states, $|{\uparrow }_{e}\,\rangle |{\Downarrow }_{h}\rangle$ and $|\,{\downarrow }_{e}\,\rangle |{\Uparrow }_{h}\rangle$ (i.e., ${\psi }_{{\uparrow }_{e}/{\downarrow }_{e}}^{e}(\overrightarrow{r})=\langle \overrightarrow{r}|{\uparrow }_{e}/{\downarrow }_{e}\rangle$, ${\psi }_{{\Downarrow }_{h}/{\Uparrow }_{h}}^{h}(\overrightarrow{r})=\langle \overrightarrow{r}|{\Downarrow }_{h}/{\Uparrow }_{h}\rangle$). With symmetry breakings such as shape deformation of QD, the *e*-*h* exchange interaction arises in the QD-confined exciton and intermixes the circularly polarized exciton states, $|{\uparrow }_{e}\rangle |{\Downarrow }_{h}\rangle$ and $|{\downarrow }_{e}\rangle |{\Uparrow }_{h}\rangle$^56^. Thus, the exciton eigenstates turn out to be $|{X}_{\pm }\rangle =\frac{1}{\sqrt{2}}(|{\uparrow }_{e}\rangle |{\Downarrow }_{h}\rangle \pm |{\downarrow }_{e}\rangle |{\Uparrow }_{h}\rangle )$, with the eigenenergies split by a fine structure splitting, denoted by *S* as presented in Fig. 1(a). Since the *e*-*h* exchange interaction is typically much smaller than the direct one, we evaluate the fine structure splitting of the exciton doublet by using the degenerate perturbation theory, which yields $S=2{V}_{{\uparrow }_{e},{\Downarrow }_{h},{\Uparrow }_{h},{\downarrow }_{e}}^{ehxc}$, where

4 $${V}_{{\uparrow }_{e},{\Downarrow }_{h},{\Uparrow }_{h},{\downarrow }_{e}}^{eh,xc}\equiv \int \,\int \,{d}^{3}{r}_{1}{d}^{3}{r}_{2}{\psi }_{{\uparrow }_{e}}^{e\ast }({\overrightarrow{r}}_{2}){\psi }_{{\Downarrow }_{h}}^{h}({\overrightarrow{r}}_{2}){\textstyle \tfrac{{e}^{2}}{4\pi {\varepsilon }_{0}{\varepsilon }_{b}|{\overrightarrow{r}}_{12}|}}{\psi }_{{\Uparrow }_{h}}^{h\ast }({\overrightarrow{r}}_{1}){\psi }_{{\downarrow }_{e}}^{e}({\overrightarrow{r}}_{1}),$$

is the matrix element of *e*-*h* exchange interaction^57^. Note that *S* is a signed value, whose sign indicates the order of the energy levels of *x* and *y*-polarized exciton states.

In the numerical calculation, the matrix elements of *e*-*h* exchange interactions are divided in the long-ranged and short-ranged parts according to the averaged Wigner-Seitz radius, and computed separately following the methodology in refs^46,58^. The former is treated in the dipole-dipole interaction approximation and numerically integrated using trapezoidal rules and the graphics processing unit (GPU) parallel computing technique for numerical acceleration. The latter is only considered for the matrix elements involving exciton states with equal angular momenta and evaluated using Eq. (2.17) in ref.^59^, in terms of the energy splitting between the bright- (BX) and dark-exciton (DX) states, ${E}_{X}^{S}={{\rm{\Delta }}}_{eh,bulk}^{xc}\times [\pi {({a}_{B}^{\ast })}^{3}\,\int \,{d}^{3}r|{g}_{{s}_{z}=\pm \tfrac{1}{2}}^{e}{|}^{2}|{g}_{{j}_{z}=\mp \tfrac{3}{2}}^{h}{|}^{2}]$, which is extrapolated from the BX-DX splitting ${{\rm{\Delta }}}_{eh,bulk}^{xc}=20$ *μ*eV of a pure HH-exciton (with the effective Bohr radius ${a}_{B}^{\ast }=12$ nm) in GaAs bulk^59^.

## Results and Discussion

### Electrically biased QDs

First, let us consider GaAs QDs under the electric field created by the voltage drop between a top and a back biased gate electrodes, as depicted by Fig. 1(b). To collect the light vertically emitted from the QDs, a *μ*m-scaled (circular) aperture is created in the top gate electrode. The patterned gate electrode establishes an inhomogeneous electrostatic field in the region below the aperture, which is more or less titled from the growth-direction, depending on the position with respect to the aperture boundary.

Figure 1(b) shows the finite element results for the electric field distribution underneath the patterned electrode with a circular aperture. The simulation shows that, whereas the electric field is certainly vertical right at the center of the aperture, a QD laterally displaced a few tens of nanometer from the center of the aperture experiences an electric field tilted by *θ*_*F*_ ~ 10°. The electrical field in a QD can be considered roughly constant because of small size of QD.

### Vertical bias

First, let us examine the energy spectrum of a QD located at the center of the electrode aperture, which is subject to a exactly vertical electric field. Since the overall tilt of the electric field in the area under the aperture is slight (|*θ*_*F*_| ≤ 20°), the single-particle spectra of the QD at the center of aperture can be regarded as a representative possessing the generic features of the energy spectra of QDs everywhere in the zone of interest. In fact, our numerical simulations show that a small tilt of electric field does not significantly change the single particle spectra, but do impact the *μ*eV-scaled exciton FSSs of QDs, as presented later.

**Figure 2:**
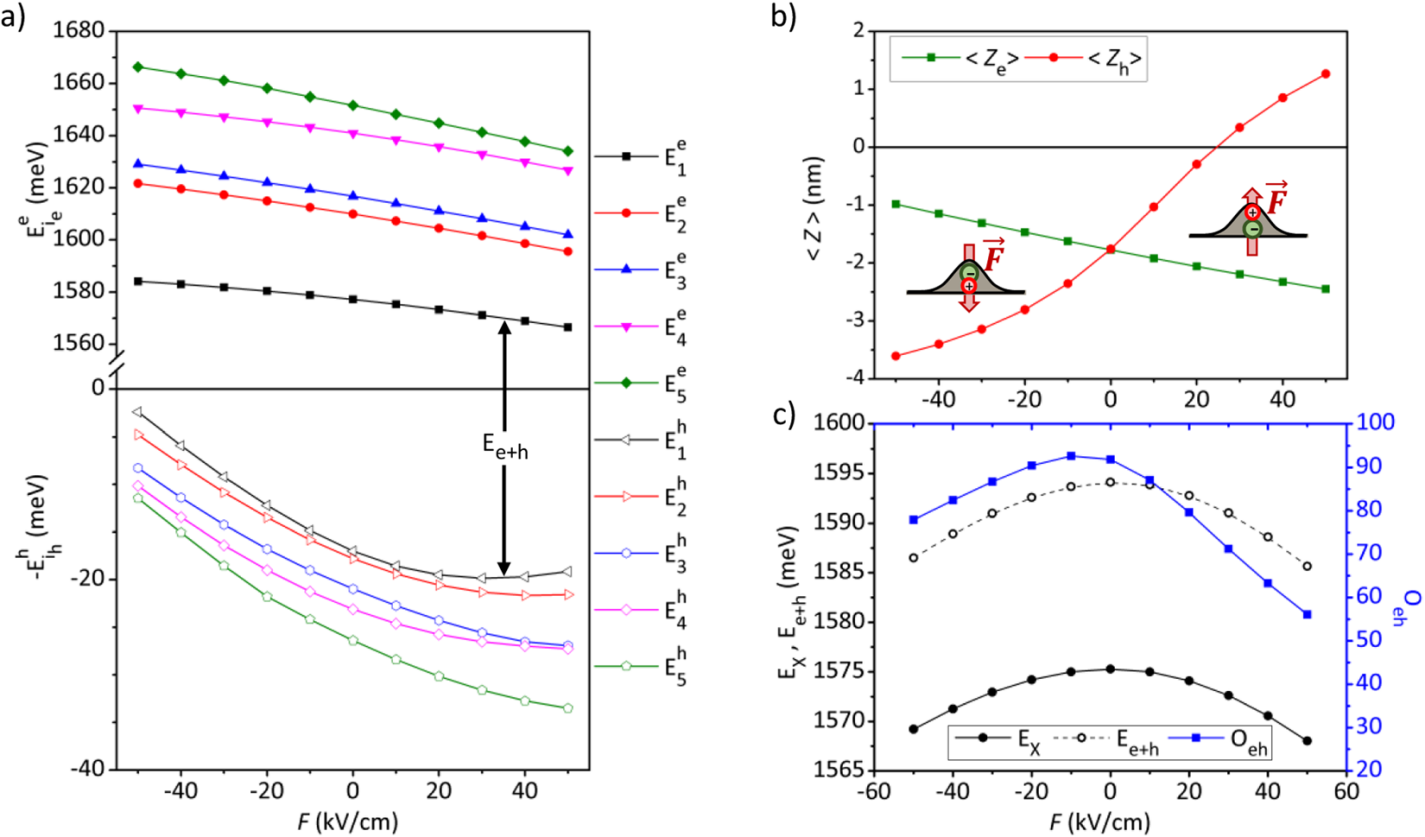
(**a**) Calculated energies of the low-lying single-electron (top) and single-hole levels (bottom) of the biased GaAs DE-QD described in the main text. (**b**) mean values of the coordinate positions of the electron (green) and hole (red) wave functions in the *z*-direction. (**c**) Calculated energies of the ground states of an exciton (black filled circles) and the overlap between electron and hole wave functions (blue squares) for the biased QD. For comparison, the total kinetic energy of the QD-confined exciton without the renormalization of the exciton binding energy is plotted and represented by the black unfilled circles. All quantities are plotted as functions of the vertical bias field.

Figure 2(a) presents the calculated eigenenergies of the five lowest single electron and hole eigenstates, as functions of vertical bias field along the growth direction. By sweeping the bias field from negative to positive, the energy of the conduction electron levels, ${E}_{i}^{e}$ decrease while those of the valence hole ones, ${E}_{i}^{h}$, increase. The asymmetric and opposite trends of the electron and hole levels in the Stark effects are associated with the fact that the Gaussian-function-profiled DE-QD lacks the up/down mirror symmetry in the shape geometry. With a positive bias field, the confined negatively charged electron in the QD is pushed down towards the bottom side of QD where the lateral confinement is weaker, whereas the positively charged hole is pulled up to the top side subject to stronger lateral confinement. It turns out that a positive bias field causes the decrease (increase) of the energies of the conduction electron (the valence hole) levels. The averaged *z*-positions of the electron and hole wave functions confined in the QD versus the applying vertical bias are plotted in Fig. 2(b).

In the Stark effect, the field-driven movements of the electron and hole in the opposite *z*-direction lead to the reduced spatial overlap between the electron and the predominant heavy-hole envelope wave functions, ${O}_{eh}\equiv \langle {g}_{1}^{e}|{g}_{1,{j}_{z}=3/2}^{h}\rangle$. Figure 2(c) shows the energy of the exciton ground state, ${E}_{X}\equiv {E}_{-}^{X}$, and *O*_*eh*_ of the QD as functions of the applying vertical electrical field. The latter exhibits a noticeable asymmetry with respect to the field direction (upward or downward) because of the vertical asymmetry of the QD^60–62^. Though the reduced *e*-*h* wave function overlap under a bias leads to the reduction of the exciton binding energy, the exciton energy of the biased QD is dominated by the total kinetic energy of electron and hole and shown decreased by both positive and negative fields. Concerned with the brightness of emitted photons from QDs, we are mainly interested in the moderate field-regime, *F* = {−30, +30} kV/cm, where the degree of *e*-*h* wave function overlap is as high as *O*_*eh*_ > 70%.

**Figure 3:**
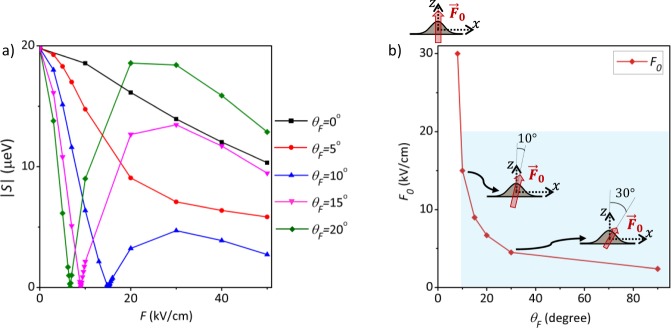
(**a**) Calculated magnitudes of the exciton fine structure splittings (|*S*|) of a GaAs DE-QD under titled electrical fields in the *x*–*z* plane with different tilting angles, *θ*_*F*_ = 0°, 5°, … 20°, with respect to the *z*-axis. One sees that, with *θ*_*F*_ > 10°, *S* can be fully suppressed by critical electric fields of moderate strengths (*F*_0_ < 20 kV/cm). (**b**) Magnitudes of the critical tilted fields *F*_0_ as a function of *θ*_*F*_. The blue shaded region highlights the cases whose *S* can be suppressed by the critical fields, *F*_0_ < 20 kV/cm.

The calculated FSSs between polarized exciton states of the QD under electrical fields with different inclinations are plotted in Fig. 3(a). With the *vertical* (*θ*_*F*_ = 0°) electric field increasing from 0 kV/cm up to 30 kV/cm, the magnitude of the excitonic FSS of the elongated QD is decreased from *S* = 20 *μ*eV to 14 *μ*eV, but still remains much higher than the criteria ($S\lesssim 1$ *μ*eV) for generating entangled photon pairs. Decomposing the hole wave function into the heavy- and light-hole components and examining the numerically calculated *e*-*h* exchange interaction of Eq. 4, it is shown that the FSS of the relatively large (as compared with InAs QDs grown in the Stranski-Krastanov mode) GaAs DE-QD studied here, is dominated by the long-ranged part of the *e*-*h* exchange interaction between the electron and heavy-hole wave functions. Thus, the magnitude and sign of the *S* of a DE-QD is mainly associated with the degree of elongation of the heavy-hole envelope wave function.

### Tilted Bias

We now proceed with the simulation of the FSS of a GaAs QD under tilted electrical fields. As shown in Fig. 3(a), the FSS of the elongated QD turns out to decrease with increasing the field tilting angle *θ*_*F*_ in the *x*-*z* plane. As *θ*_*F*_ > 10°, the FSS of the elongated QD can be vanishing and cross over *S* = 0 at a specific bias field, *F*_0_. Figure 3(b) plots the magnitude of *F*_0_ versus the tilting angle of the electric field. One sees that, the greater the tilting angle, the smaller the necessary field to achieve vanishing *S*. With a small tilting angle, *θ*_*F*_ ~ 10°, the required magnitude of electric field for making *S* = 0 is below 20 kV/cm. Thus, a small in-plane component in an applied bias field onto a QD could make a key contribution to the suppression of the FSS of an elongated QD by electrical means. The electric-field-driven reduction of the FSS of the *x*-elongated QD under the tilted field results from the decreased long-ranged part of the *e*-*h* exchange interaction, which is due to the combined effect of the smaller spatial overlap of electron and hole wave functions and the reduced degree of elongation of the exciton wave function by the titled field. Here, it is the latter effect that predominantly suppresses the FSS of the elongated QD down to *S* ~ 0.

**Figure 4:**
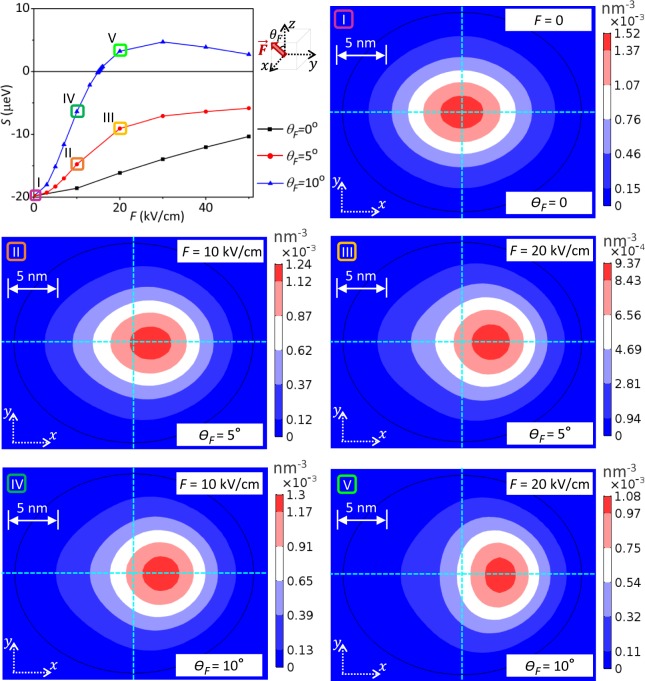
Calculated charge density of the leading heavy-hole component of the exciton wave function for the QD under different electrical fields. The left-topmost panel replots, for the particular tilting angles 0°, 5°, and 10°, the electric-field dependent *S* of the biased QD considered in Fig. 3(a). Panels (I–V) show five representative cases that are selected to examine the corresponding field-dependent hole density distributions, in the plane (*x*, *y*, *z* = *h*/3) of the QD. One observes from the cases IV and V that the sign of *S* is switched from negative to positive as the magnitude of the field slanted by an angle *θ*_*F*_ = 10°, increases. Correspondingly, the hole wave function is changed, from *x*-elongated (case IV), to be *y*-elongated (case V).

To elucidate that, Fig. 4 presents the field-dependent charge density in the *x*–*y* plane of the leading heavy-hole component of the exciton wave function for the QD under different slightly tilted electrical fields (*F* = 0, 10, 20 kV/cm and *θ*_*F*_ = 5°, 10°). The *S* corresponding to each of the applied electrical fields are indicated in Fig. 4(a). Note that the sign of *S* is electrically reversible as the field is properly tilted. Because of heavier mass, the hole wave function is more localized in the QD than that of electron, and dominates the spatial distribution of the exciton wave function. Thus, here we can examine the hole wave function to grasp how, in the sense of symmetry, the exciton wave function is reshaped by external electrical field and the sign of FSS reversed.

The panel I of Fig. 4 plots the hole charge density for the QD without electrical bias, which shows slightly elongated along the *x*-direction simply due to the *x*-elongated QD-confinement. It is the *x*-elongation of wave function that gives rise to the long-ranged *e*-*h* exchange interactions and leads to the fine structure splitting, *S* ~ −20 *μeV*. Applying an electric field titled from the *z*-axis towards the *x*-axis deforms the wave function, i.e. reducing the degree of *x*-elongation but increasing that along the *y*-axis, and attenuates the long-ranged *e*-*h* exchange interactions and the magnitude of the resulting *S* as well. Increasing the tilting angle of field makes larger the in-plane field component and the drop of |*S*| is more pronounced even to to the degree of reversing the sign of *S*. As highlighted in the top-left panel of Fig. 4, the splitting *S* between *F* = 10 kV/cm and *F* = 20 kV/cm undergoes a sign reversal for *θ*_*F*_ = 10°, since the wave function elongation is switched from the *x*-axis to the *y*-one by the titled field, as seen by comparing the panels I and V of Fig. 4.

Besides the advantageous effect on the FSS-reduction, tilting a vertical field might decrease the overlap of the electron and hole wave functions, and weaken the optical brightness of the QD emitters. Our simulation shows that the wave function overlap is decreased by ~8% as *θ*_*F*_ = 10° and *F* = 10 kV/cm. The decrease of the wave function overlap is enlarged roughly linearly with the increasing tilting angle, but can be reduced by increasing the magnitude of *F*. Thus, using a high electric field that is titled appropriately (*θ*_*F*_ < 20°) should make the optimal effect on the QD emitter.

**Figure 5:**
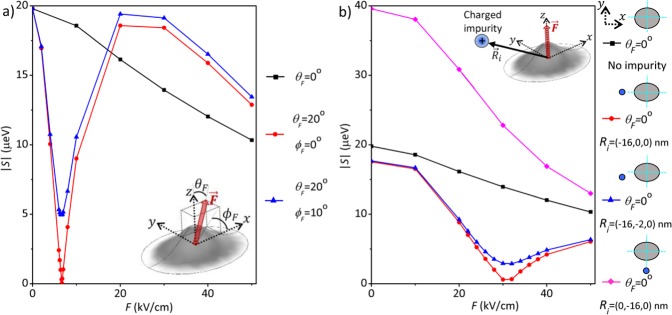
(**a**) Magnitudes of the fine structure splittings, |*S*|, versus the strengths of the applied bias, for the GaAs DE-QD under differently directed electric fields. Ideally vertical field (black squares), field tilted within the *x*–*z* plane (blue triangles), and field lying out of the *x*–*z* plane (red circles). (**b**) Calculated *S*, versus the applied vertical electric field, of the GaAs DE-QD under the additional influence of a single charged impurity positioned at different locations.

Considering generic electric fields under the aperture of electrode, Fig. 5(a) presents the calculated *S* for the QD under an electric field misaligned from all principal axes, which is titled from the *z*-axis by *θ*_*F*_ = 20° and from the *x*–*z* plane by the angle *ϕ*_*F*_ = 10°. For comparison, the previously studied cases of vertical electric field (*θ*_*F*_ = 0° and ${\varphi }_{F}=0^\circ$) and the titled field in the *x*–*z* plane (*θ*_*F*_ = 20° and *ϕ*_*F*_ = 0°) are presented in the figure as well. In the currently studied case (*θ*_*F*_ = 20° and *ϕ*_*F*_ = 10°), the presence of the in-plane field component perpendicular to the elongation axis undermines the ability of the bias field for undoing the lateral asymmetry and no longer can thoroughly suppress the FSS of the QD. Nevertheless, as compared with the case of exactly vertical field, both titled fields show enhanced ability to tune the FSS of the dot. i.e. the FSS of the QD drops by ~15 *μ*eV (from 20 *μ*eV to 5 *μ*eV) with the application of the tilted field, *F* ~ 10 kV/cm, while the decrease of FSS in only about 2 *μ*eV with the perfectly vertical field of the same strength.

### Effects of charged defects or impurities

Regarding the electrostatic environment experienced by QDs, charged impurities or defects could make a certain impact on the optical and excitonic properties of the dots^27^. Electrostatic modifications to the confinement potential by charged defects at the QD vicinity, have been recently discussed and characterized in self-assembled dots^25,26^, and DE dots^27^. Without losing generality, we consider a single point charge located at the vicinity of the biased dot and investigate its influence on the FSS. Figure 1(c) depicts a studied case where a positive point charge is positioned at 16 nm from the dot center. In addition to the electric field arising from the point charge, the finite element simulations reveal that the strength of the combined electric field passing through a QD under the aperture is ranged between *F* = 35–70 kV/cm, with the tilting angle varied from *θ*_*F*_ = 15° to 55°.

Figure 5(b) shows the calculated fine structure splitting, *S*, of the QD under the combined influence of an applied vertical electrical bias and a positive charge point located in different sites on the QD plane (the impurity-dot distance is maintained constant at 16 nm). Let us first consider the point charge sitting at the *x*-axis. As seen in Fig. 5(b), the FSS of the QD under the influence of the point charge at the axis of QD-elongation can be even suppressed by a vertical field of *F* ~ 30 kV/cm. In that case, the point charge provides an in-plane field component that is similar to that of a tilted field alone in the *x*–*z* plane (compare the both red lines of Fig. 5(a,b)) and tends to regain the symmetry of the exciton wave function that can lead to *S* = 0. Moving the point charge away from the *x*-axis, the suppression of the FSS of the QD is diminished (See the blue and magenta lines in Fig. 5(b)). As the point charge is moved to the *y*-axis, it turns out that the FSS of the QD is even magnified, which is ranged from 40 *μ*eV at *F* = 0 to 14 *μ*eV at *F* = 50 kV/cm and overall greater than that of QD in the absence of any point charge. An interesting observation is that, no matter where is the position of the point charge, the electrostatic effect of point charge is to raise the FSS electrical tunability. We see in Fig. 5(b) that, in the presence of a point charge, the FSS of the QD drops 3–4 times faster with increasing the vertical bias from *F* > 10 kV/cm than that of the QD without any point charge nearby.

Additionally, the black, blue and red curves in Fig. 5(a,b), evidence that the efficiency in reducing the FSS by means of electric field, may vary noticeably from dot to dot (even if they are identical), because of differences in the field directions or charged environment. Resemblant features have been experimentally observed in samples containing dots expected to be very similar [see Fig. 3(c) of reference^22^]. Therefore, this may explain the puzzling diverse FSSs of dots with alike sizes, shapes and growth conditions.

### Impacts of electrostatic environment

In reality, the precise tilting angle of the resulting electrical fields from biased electrodes and residual charges underneath the sample surface is hardly known and mostly out of control. The factor of electrical bias is determined not only by the pattern- and layer-structures of electrodes and semiconductors but also dependent on the exact locations of the QDs, that are essentially random and unknown. On the other hand, the presence of residual charges in the QD environment is in principle arbitrary, inevitable and even dynamic^63^. Yet, the simulation results of Fig. 5 reveals that the FSSs of QDs are dramatically impacted by the presence of any unexpected small in-plane component of electric field. Examining all the studied cases of Fig. 5, the magnitude of the FSS of the QD could be widely varied between 0 and 40 *μ*eV, two times the magnitude of the FSS of the QD subject to no field. Thus, one realizes from the investigation that, besides the intrinsic differences in the structures (size, geometry, composition, etc.) of QDs, the electrostatic environment that is particularly experienced by each QD should be another important factor underlying the statistical randomness and scattering of the measured FSSs for a QD ensemble sample, restricting the yield of successful QD-based entangled photon pair emitters. So far, the electrostatic factors seem to be considered as obstacles for optimal FSS control. However, one might take advantage of our improved understanding of the electrostatic effects to increase the efficiency of QD-based quantum emitters with appropriate design of devices. As we realize that a small in-plane component of a slightly tilted electric field could be helpful for minimizing the FSS of a QD if that component lies along the elongation-axis of QD. It has been well established that, in spite of self-assembling growth, the shapes of self-assembled QDs are naturally elongated along a specific crystalline axis. For instance, GaAs DE-QDs on (001) substrates are naturally elongated in the $[1\bar{1}0]$ direction^28^. Based on the fact, elliptical apertures (sharing elongation axis with the dots) instead of circular ones, or a multi-finger structure of electrode with independently individual bias controls over each finger electrodes can be used to purposely produce the in-plane field components along the QD elongation. The feasibility of the idea of using multiple controlled biases is supported by the recent success in the deterministic production of entangled photon pairs from InAs self-assembled QDs that is achieved by means of using at least two independent control knobs (electric, stress, or magnetic fields) for the FSS-tuning^11,41,64^.

## Conclusions

In conclusion, we presented a theoretical and computational investigation on the excitonic fine structures of GaAs/AlGaAs DE-QDs under the influence of the electrical fields established by gating electrodes and surrounding charged impurities within the scheme of a multi-band theory, under realistic considerations for the QD-confining potential geometry and the electron-hole Coulomb interactions. The computed results show that the feasibility of electrically-triggered entangled photon pair generation from of a DE-QD in the vertical bias configuration could be largely impacted by the presence of an unexpected in-plane component of electric field due to a non-central location of QD with respect to the patterned electrodes and/or residual charged defects or impurities near the QD. As a minimum impact on the FSS of a QD, the presence of a single charged impurity sitting right beside the QD is shown to increase the FSS of the QD by a double of the magnitude or, oppositely, decrease it down to be nearly zero, depending on the relative position of the charged impurity to the elongated QD. Even in the absence of any charged impurities or defects, a slight tilting angle of the applied electrical field (say *θ*_*F*_ = 10° and *F* = 20 kV/cm) onto a QD would make substantial changes in the FSS of the polarized exciton doublet by a magnitude comparable to the FSS itself and even reversing the sign of FSS (i.e. reordering the order of the spin-split polarized exciton doublet levels in energy). The investigation accounts for that, besides the inherent structural differences among QDs in a sample (size, geometry, composition, etc), the electrostatic environment that is experienced by each individual QD should be another important factor behind the statistical randomness and scattering of the measured FSSs for QDs in an ensemble sample, restricting the yield of successful QD-based entangled photon pair emitters. Remarkably, the extrinsic electrostatic effects revealed by this study do not always just make an obstacle for the optimal control over the FSSs of QDs towards the realization of entangled photon pair emitters with deterministic removal of the FSS. It is shown that a small horizontal component, lying in the plane comprising the elongation axis (perpendicular to the growth direction), of an tilted electric field is particularly effective to suppress the FSS of an elongated QD. Even an tilted field not perfectly lying in but close to the specific plane can greatly enhance the FSS-tunability and is advantageous in the electrically triggered entangled photon pair production from QDs. The improved understanding of the electrostatic effects on the FSSs of DE-QDs provided by this study offers an useful guidance for the optimal design and fabrication of the QD-based quantum emitters. As we recognize the advantageous effect of in-plane field component, a multi-finger structure of electrode could be considered in the fabrication of electrically operated QD-based quantum emitters to purposely produce a tilted electrical bias field, composed of the principal vertical bias and the independently controlled in-plane field components along the QD elongation, for optimizing the production of entangled photon pairs.
